# Intestinal barrier function as a key determinant of inflammation and nutritional status in digestive surgery patients: a real-world study

**DOI:** 10.3389/fnut.2025.1637877

**Published:** 2025-09-05

**Authors:** Jingjing Wang, Jing Yan, Linlin Shi, Ying Wang, Xiaoxiao Tian, Yumei Qi, Guoxun Li

**Affiliations:** ^1^Department of Nutrition, The Third Central Hospital of Tianjin, National Medical Quality Control Center of Clinical Nutrition, Tianjin Key Laboratory of Extracorporeal Life Support for Critical Diseases, Artificial Cell Engineering Technology Research Center, Tianjin Institute of Hepatobiliary Disease, Tianjin, China; ^2^Department of Nutrition, Tangdu Hospital, Fourth Military Medical University, Xi’an, China

**Keywords:** intestinal barrier, nutrition treatment, inflammation, digestive surgery patients, early enteral nutrition

## Abstract

**Introduction:**

Existing studies have demonstrated a significant correlation between intestinal barrier and disease outcomes. The intestinal barrier is particularly susceptible to impairment following digestive surgery. The study aimed to elucidate the effects of intestinal barrier impairment on inflammation and nutritional status, as well as the necessity of nutritional treatment for postoperative patients.

**Methods:**

We assessed intestinal barrier integrity by measuring serum biomarkers, diamine oxidase (DAO), D-lactate (D-lac) and lipopolysaccharide (LPS) in 745 consecutive hospitalized patients after digestive surgery and 394 non-surgical patients. Serum levels above established cutoffs (DAO > 10 U/L, D-lac >15 mg/L, LPS > 20 U/L) were defined as positive, corresponding to mucosal injury, increased intestinal permeability, and bacterial translocation. Correlation analyses were performed between intestinal barrier integrity, inflammation, cytokines, and nutritional status. The areas under the receiver operating characteristic (ROC) curves were used to predict severe intestinal barrier impairment. Additionally, changes in intestinal barrier biomarkers were compared after 1 week of nutritional therapy.

**Results:**

Postoperative patients exhibited a high incidence of intestinal barrier impairment. Among the biomarkers, DAO showed the highest positivity rate, followed by D-lac, while LPS was the least frequently elevated. The highest levels of serum DAO, D-lac and LPS were observed in patients with severe intestinal barrier impairment (positive for all three biomarkers). Patients with intestinal barrier impairment exhibited progressively worsening nutritional status and escalating systemic inflammation. The area under the ROC curve for predicting severe intestinal barrier impairment was 0.71. One-week nutritional intervention was significantly associated with improved intestinal barrier function, primarily evidenced by a reduction in intestinal permeability. Early enteral nutrition (EEN) was associated with lower serum DAO, D-lac, and LPS levels. However, patients with aggravated intestinal barrier function after nutritional therapy displayed higher inflammatory markers and failed to achieve improvement in nutritional status compared to those with improved barrier function.

**Conclusion:**

Intestinal barrier impairment is prevalent in patients undergoing digestive surgery and acts as a key driver of both inflammation and malnutrition. EEN was associated with improvement in intestinal barrier dysfunction. However, delayed or inadequate correction of intestinal barrier impairment may compromise therapeutic outcomes.

## 1 Introduction

The gastrointestinal tract serves not only the central organ for digestion and absorption but also the primary defense against the invasion of pathogens and toxins. The intestinal barrier, composed of mechanical, chemical, immune, and biological barriers, is crucial for maintaining intestinal homeostasis (1, 2). Recent studies have further highlighted the role of gut in immunity, accounting for approximately 70–80% of the body’s immune function (3, 4). Intestinal barrier is involved in the regulation of various systemic diseases such as diabetes (5, 6), fatty liver disease (7), cardiovascular disease (8, 9), asthma (10, 11), primary sclerosing cholangitis (12), rheumatoid arthritis (13) and infectious diseases like influenza (14) and tuberculosis (15). Undoubtedly, maintaining the normal physiological function of the intestinal barrier is fundamental to overall health.

Patients undergoing digestive surgery often experience impairment of intestinal barrier function due to surgical trauma, anesthetic stress (16), and inflammatory responses. This impairment can lead to bacterial translocation, systemic inflammatory response syndrome (SIRS), and multiple organ dysfunction, significantly affecting disease outcomes (17, 18). However, in current clinical practice, a dynamic monitoring for postoperative intestinal barrier function remains to be established, and the underlying mechanisms governing the interplay between barrier repair, nutritional metabolism, and inflammatory regulation urgently require elucidation. Clinical data show that patients with impaired intestinal barrier function experience a 30–50% higher incidence of infectious complications, along with hospital stays prolonged by more than 20%. Therefore, intestinal barrier impairment in postoperative gastrointestinal patients adversely affects clinical outcomes, urgently necessitating the development of evidence-based therapeutic interventions.

Nutritional intervention is a core strategy to improve postoperative intestinal barrier function. A great number of studies emphasize that patients with stable hemodynamics should initiate EN promptly to maintain the integrity of the intestinal mechanical and immune barriers (19). Postoperative protein-energy malnutrition can deplete essential nutrients such as glutamine (Gln) (20, 21) and short-chain fatty acids (SCFA) (22, 23), which are critical for the proliferation of intestinal mucosal epithelial cells. This deficiency can lead to villus atrophy and the down-regulation of tight junction protein expression. However, the optimal timing and protocol for initiating enteral nutrition (EN) in post-gastrointestinal surgical patients remain controversial. While previous studies have predominantly focused on the effects of individual nutrients on intestinal barrier function, the systemic impact of comprehensive nutritional strategies has been largely overlooked. This prospective observational study conducted in real-world clinical settings systematically assessed intestinal barrier integrity among surgical patients receiving nutritional care encompassing combined parenteral and/or enteral approaches, all achieving predefined nutritional targets within the initial postoperative week. The investigation specifically compared clinical outcomes between two treatment strategies—one receiving early enteral nutrition (EEN) supplementation alongside parenteral nutrition (PN) support and the other maintained on complete fasting status with exclusive PN. The research objectives centered on elucidating the capacity of EEN to facilitate intestinal barrier restoration while concurrently analyzing the reciprocal regulatory mechanisms linking barrier functional recovery trajectories to the dynamic evolution of nutritional status indicators.

Intestinal barrier impairment has gradually become a focal point in clinical research and practice. Several biomarkers are utilized to evaluate intestinal barrier function in patients, with DAO, D-lac and LPS being the most commonly employed. DAO reflects intestinal mucosal integrity, as it is primarily synthesized by mature enterocytes. Elevated serum DAO indicates epithelial damage, as the enzyme is released into circulation during cell shedding (24). D-lac, a byproduct of bacterial metabolism, enters the bloodstream only when intestinal permeability is increased, making it a direct marker of barrier leakage (25). Lipopolysaccharide (LPS), a component of gram-negative bacterial membranes, translocate systemically during barrier disruption, triggering inflammation and serving as an indicator of microbial translocation (26). These biomarkers are widely recognized for their relatively high sensitivity and specificity, while maintaining non-invasive collection protocols suitable for routine practice (27, 28).

The present study aims to systematically analyze the characteristics of intestinal barrier dysfunction in postoperative digestive surgery patients and its complex interactions with nutritional status and inflammatory responses, thereby providing a scientific basis for clinical practice and promoting the continuous advancement of intestinal barrier protection strategies and nutritional therapy approaches.

## 2 Materials and methods

### 2.1 Study population and data collection

**Figure 1:**
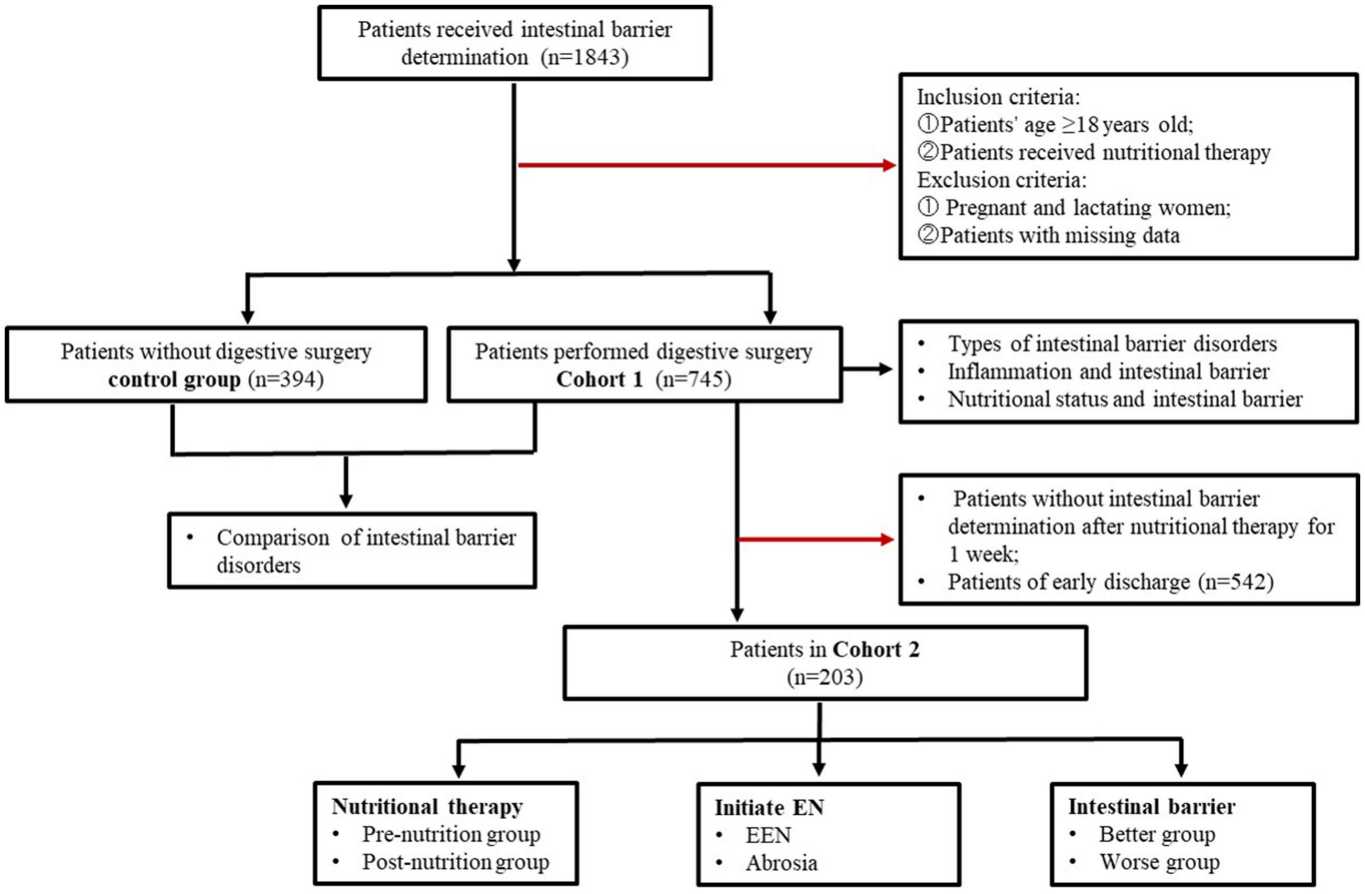
Flow diagram for the selection of patients in the study.

A total of 1,843 inpatients received intestinal barrier determination were enrolled from Tianjin Third Central Hospital between September 2021 and July 2024. The flow of patient selection and exclusion was illustrated in Figure 1. In Cohort 1, a total of 745 patients who underwent digestive surgery received nutritional therapy and had their intestinal barrier function determined on the first day after surgery. The control group included 394 patients who did not undergo digestive surgery. Cohort 2 included 203 patients who underwent intestinal barrier determination on the first and the seventh day after surgery. The study was approved by the Ethics Committee of Tianjin Third Central Hospital (No. IRB2024-01-0003).

### 2.2 Nutritional therapy procedure

All adult inpatients underwent nutritional risk screening based on Nutrition Risk Screening (NRS) 2002 within 24 h of admission. Postoperative patients were performed NRS 2002 screening again on the first day after surgery. If the NRS 2002 score was ≥3, a nutritionist conducted a comprehensive nutritional assessment and initiates appropriated nutritional therapy. Nutritional treatment was administered via the appropriate route. Enteral nutrition (EN) was the preferred option, whereas parenteral nutrition (PN) was administered to patients who cannot tolerate EN. EN treatment included oral diet, oral nutritional supplement (ONS), gastric tube nutrition and jejunum tube nutrition. Oral diets are categorized into liquid, semi-liquid, and solid foods. Early enteral nutrition (EEN) is defined as the initiation of gastrointestinal feeding within 48–72 h postoperatively. Nutritional prescriptions were adjusted daily, with a total energy target of 25–30 kcal/kg/day including both EN and PN. PN was provided as an all-in-one admixture containing compound amino acid injection (18AA), lipid emulsion (MCT/LCT), glucose, electrolyte solution (sodium chloride injection, potassium chloride injection, calcium carbonate injection, magnesium sulfate injection, sodium glycerophosphate injection), trace elements (Multi-trace Elements Injection II; FRESENIU KABI SSPC, China), water-soluble vitamins (Verapamil Hydrochloride Tablets; FRESENIU KABI SSPC), and lipid-soluble vitamins (Fat-soluble Vitamin Injection II; FRESENIU KABI SSPC). Nutritional supplementation was gradually increased for patients postoperatively with the target energy intake achieved by the seventh day.

### 2.3 Blood biochemistry and intestinal barrier function assessment

Blood samples were collected from patients early in the morning after an overnight fast. All samples were processed for biochemical analysis at the biochemistry laboratory in hospital. White blood cell count (WBC), neutrophil count (NE), lymphocyte count (LYM), and hemoglobin (Hb) in the blood samples were quantified on the ADVIA-2120 autoanalyzer. Prognostic nutritional index (PNI) was calculated as (ALB + 5 × lymphocyte count). Serum C-reactive protein (CRP) was measured on a light scattering turbidimeter (IMMAGE 800; Beckman). Procalcitonin (PCT) was detected by enzyme-linked fluorescence assay (ELFA) using the automatic chemiluminescence immunoassay system (VIDAS, Biomerieux, France). Blood albumin (ALB), prealbumin (PA) and retinol-binding protein (RBP) levels were measured using automatic biochemical analyzer MODULAR P800 (Roche). Serum cytokines were measured using liquid suspension chip technology (Luminex xMAP), and the instrument was Luminex 200 (Luminex, America). Intestinal barrier function was assessed by measuring serum DAO, D-lac and LPS using the JY-DLT system (Beijing Zhongsheng Jinyu Diagnostic Technology, China). The cutoff values are DAO < 10 U/L, D-lactate < 15 mg/L, and LPS < 20 U/L.

### 2.4 Statistical analysis

SPSS (version 22.0) and Graph Pad Prism (version 8.2.1) were used for data analyses and visualization. Variable data distribution was evaluated using the Shapiro–Wilk test. Normally and non-normally distributed data were expressed as means ± standard deviations and medians with interquartile ranges (IQRs), respectively; categorical variables were expressed as counts and percentages. Correlation analysis was conducted using a Pearson’s correlation coefficient and simple linear regression. The difference between groups was performed by *t*-test. One-way analysis of variance (ANOVA) was used for multiple samples. SNK test was performed for multiple comparisons between groups. To evaluate the predictive power and the diagnostic performance for severe intestinal barrier impairment, receiver operating characteristic (ROC) curve analysis was performed, and the area under the ROC curve (AUC) was calculated. *p* < 0.05 was considered to indicate statistical significance.

## 3 Results

### 3.1 Patients characteristics

Table 1 delineates the baseline characteristics of the study population in cohort 1. A total of 745 patients were included in this study, with 68.86% were aged over 65 years. All participants underwent various forms of digestive surgery, including stomach surgery (*n* = 91, 12.22%), intestinal surgery (*n* = 233, 31.28%), hepatobiliary surgery (*n* = 223, 29.93%), and pancreatic surgery (*n* = 198, 26.58%).

**Table 1:** Patient characteristics in cohort 1.

Characteristic (*n* = 745)	Value
Age, years, x– ± *s*	67.43 ± 14.11
<45 years, n (%)	55 (7.38)
45–65 years, n (%)	177 (23.76)
>65 years, n (%)	513(68.86)
Sex, n (%)	
Female, n (%)	224 (30.07)
Male, n (%)	521 (69.93)
Surgery types, n(%)	
Stomach surgery	91 (12.22)
Intestinal surgery	233 (31.28)
Hepatobiliary surgery	223 (29.93)
Pancreatic surgery	198 (26.58)
Postoperative diet, n(%)	
No oral intake	287 (38.52)
EN	458 (61.48)

EN, enteral nutrition, including liquid diet, semi-liquid diet and oral nutritional supplements (ONS), nasogastric tube nutrition and jejunal nutrition.

Nutritional assessment indicated that 38.52% (*n* = 287) of patients were unable to tolerate oral intake, whereas 61.48% (*n* = 458) successfully commenced enteral nutrition (Table 1).

### 3.2 Digestive surgery impaired patients’ intestinal barrier function

**Figure 2:**
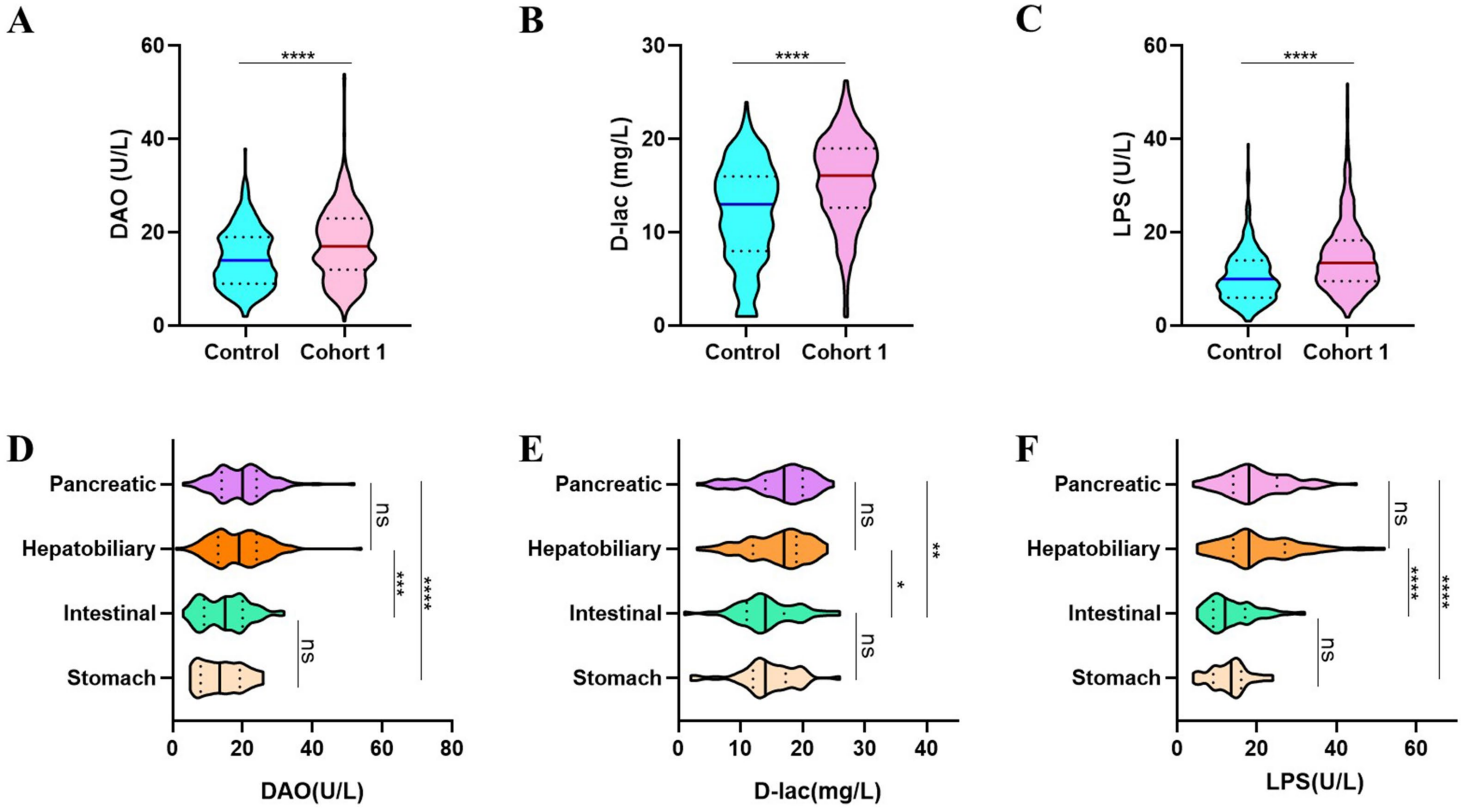
The intestinal barrier function compromised following digestive tract surgeries. **(A–C)** Patients after digestive surgery demonstrate significantly higher DAO **(A)**, D-lac **(B)** and LPS **(C)** levels. **(D–F)** Patients in Cohort 1 undergoing different types of digestive surgeries showed varying degrees of intestinal barrier dysfunction. Control, non-operated patients (*n* = 394); Cohort 1, patients performed digestive surgery (*n* = 745).

Postoperative patients who underwent digestive surgery demonstrated significantly elevated levels of intestinal barrier injury biomarkers compared to those in non-surgical controls with particularly notable increases in serum diamine oxidase (DAO, 14.48 ± 6.172 U/L vs. 17.53 ± 7.593 U/L), D-lactate (D-lac, 12.04 ± 5.271 mg/L vs. 15.95 ± 4.427 mg/L), and lipopolysaccharide (LPS, 10.48 ± 5.404 U/L vs. 16.01 ± 8.156 U/L) levels (Figures 2A–C). The extent of intestinal barrier impairment differed significantly among patients underwent various digestive surgical procedures. While gastric and intestinal surgeries induced comparable levels of intestinal barrier disruption, similar patterns were observed in hepatobiliary and pancreatic interventions. Notably, postoperative analysis revealed significantly elevated levels of intestinal barrier damage biomarkers—DAO, D-lac, and LPS—in patients following hepatobiliary and pancreatic procedures compared to those undergoing gastrointestinal surgeries (Figures 2D–F).

### 3.3 Analysis of intestinal barrier impairment in postoperative patients

**Figure 3:**
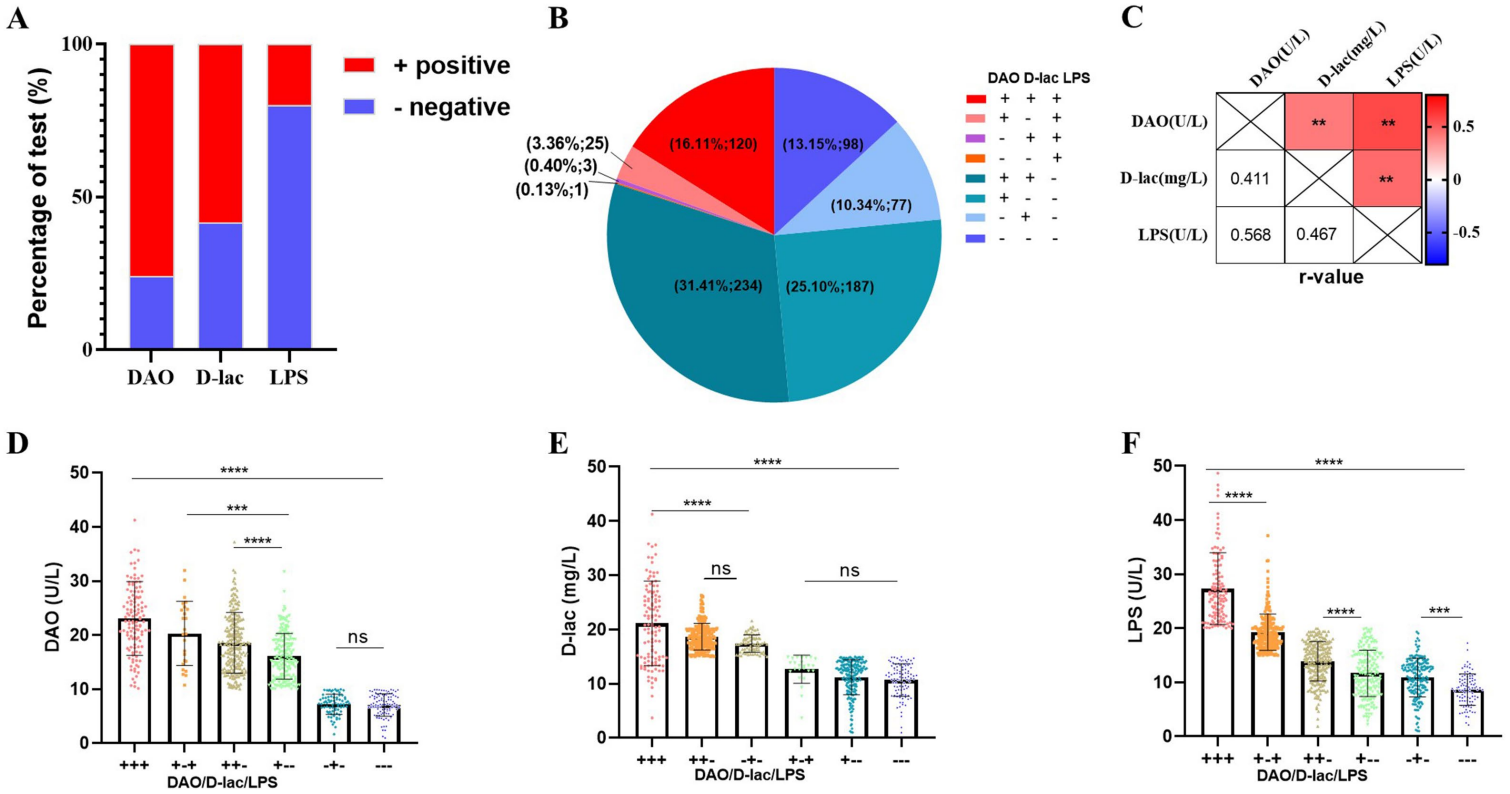
Types of intestinal barrier impairment in Cohort 1. *n* = 745. **(A)** Percentage of serum DAO, D-lac, LPS positive. **(B)** The number and proportion of patients with different type of intestinal barrier impairment. **(C)** Correlation analysis of DAO, D-lac and LPS. Pearson *r* = (−1, 1). **(D–F)** Levels of serum DAO, D-lac and LPS in patients with different types of intestinal barrier impairment. Reference Range of intestinal barrier: Serum, adult: DAO<10 U/L, D-lac<15 mg/L, LPS<20 U/L.+, above the upper limit of the reference value range; −, within the reference value range; +++, DAO>10 U/L, D-lac>15 mg/L, LPS>20 U/L; + −+, DAO>10 U/L, D-lac<15 mg/L, LPS>20 U/L; ++−, DAO>10 U/L, D-lac>15 mg/L, LPS<20 U/L; + − -, DAO>10 U/L, D-lac<15 mg/L, LPS<20 U/L; − + −, DAO<10 U/L, D-lac>15 mg/L, LPS<20 U/L; −--, DAO<10 U/L, D-lac<15 mg/L, LPS<20 U/L.

The incidences of intestinal barrier impairment in postoperative patients were high, with a rate of 75.97% for intestinal mucosal impairment (DAO+), 58.26% for increased intestinal permeability (D-lac+), and 20% for bacterial translocation (LPS+; Figure 3A). The incidence of bacterial translocation in the absence of either intestinal mucosal injury or increased intestinal permeability (+ − +/−++/−−+/) was low with 3.36, 0.40 and 0.13%, respectively, (Figure 3B). DAO, D-lac, and LPS showed positive correlations in pairwise comparisons, with correlation coefficients of 0.41, 0.47, and 0.57, respectively (Figure 3C). Furthermore, concurrent intestinal mucosal injury with either increased permeability or bacterial translocation resulted in significantly elevated serum DAO levels. Similarly, serum D-lac concentrations were higher when increased permeability coexisted with intestinal mucosal injury or bacterial translocation compared to isolated permeability alterations. Serum DAO, D-lac and LPS levels were highest in severe intestinal barrier impairment (+++; Figures 3D–F).

### 3.4 Correlation analysis of intestinal barrier impairment

**Figure 4:**
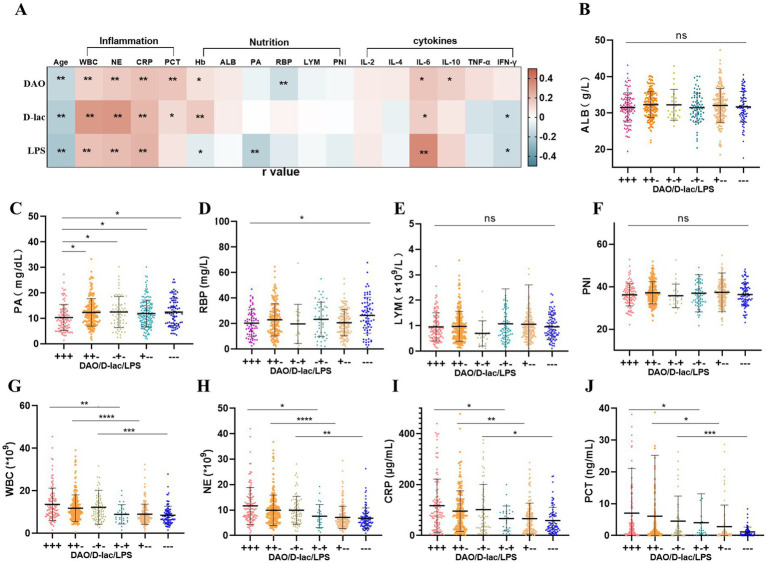
Association between intestinal barrier and inflammation and nutrition status in Cohort 1. *n* = 745. **(A)** Correlation analysis of intestinal barrier impairment. Pearson *r* = (−1, 1). **(B–F)** Nutritional status in different types of intestinal barrier impairment. **(G–J)** Inflammation in different types of intestinal barrier impairment. +, above the upper limit of the reference value range; −, within the reference value range. WBC, blood white blood cell count; NE, blood neutrophil count; CRP, C-reactive protein; PCT, procalcitonin; ALB, blood albumin; PA, blood pre-albumin; RBP, retinol-binding protein; PNI, Prognostic nutritional index; LYM, blood lymphocyte count.

We conducted an analysis to explore the correlations between the intestinal barrier impairment and multiple clinical parameters, including age, systemetic inflammation markers, nutritional status, and cytokine levels. The analysis indicated that the serum levels of intestinal barrier markers (DAO, D-lac, and LPS) were positively correlated with inflammatory indicators, with correlation coefficients ranging from 0.11 to 0.32. Conversely, inverse relationships were observed between these biomarkers and age, with correlation coefficients ranging from −0.16 to −0.23 (Figure 4A).

The correlation between hemoglobin levels and intestinal barrier impairment was inconsistent; while hemoglobin showed a positive correlation with DAO and D-lac, it exhibited a negative correlation with LPS. Additionally, serum levels of DAO and LPS were inversely associated with nutritional indicators RBP and PA, with correlation coefficients of −0.13 and −0.18, respectively (Figure 4A).

IL-6 levels demonstrated a positive correlation with serum DAO, D-lac, and LPS, with correlation coefficient (r) of 0.17, 0.15, and 0.36, respectively. Meanwhile, IFN-*γ* was negatively correlated with D-lac (*r* = −0.13) and LPS (*r* = −0.14). In contrast, IL-10 showed a positive correlation with DAO (*r* = 0.14; Figure 4A).

### 3.5 Patients with severe intestinal barrier impairment had poor nutritional status

Analysis of nutritional indicators among patients with different degrees of postoperative intestinal barrier impairment revealed notable trends. Patients without intestinal barrier impairment (−−−) exhibited the highest levels of PA and RBP, with mean values of 12.48 mg/dL and 26.50 mg/L, respectively. In contrast, those with intestinal barrier impairment showed reduced levels, with the most pronounced reduction observed in the severe impairment group (+++), where PA and RBP levels dropped to 10.36 mg/dL and 19.78 mg/L, respectively. Additionally, other nutritional indicators, such as ALB, LYM, and PNI, also appeared to be lowest in patients with severe intestinal barrier impairment (+++). However, no statistically significant differences were detected among the groups (Figures 4B–F).

### 3.6 Patients with severe intestinal barrier impairment had higher inflammation

Postoperative inflammation levels varied among patients with different degrees of intestinal barrier impairment. Patients without intestinal barrier impairment (−−−) exhibited the lowest levels of inflammatory markers, including WBC (8.52 × 10^9^/L), NE (6.81 × 10^9^/L), CRP (59.13 μg/mL), and PCT (2.32 ng/mL). In contrast, patients with intestinal barrier impairment exhibited a progressive increase in inflammatory markers. Among them, patients with severe intestinal barrier impairment (+++) had the highest inflammation levels, with WBC, NE, CRP, and PCT measuring 13.62 × 10^9^/L, 11.65 × 10^9^/L, 117.27 μg/mL and 7.05 ng/mL, respectively. Furthermore, increased intestinal permeability (D-lac+) was a key factor contributing to the elevation of inflammation (Figures 4G–J; Table 2).

**Table 2:** Analysis of influencing factors of intestinal barrier function impairment.

Variable	^1^Normal group *n* = 98	^2^Mild group *n* = 264	^3^Moderate group *n* = 259	^4^Severe group *n* = 120	*F* value	*p* value
DAO(U/L)	7.05 ± 2.08^d^	13.45 ± 5.49^c^	18.63 ± 5.76^b^	23.08 ± 6.82^a^	161.13^**^	<0.01
D-lac(mg/L)	10.72 ± 2.98^d^	13.02 ± 4.0^c^	18.05 ± 3.02^b^	20.03 ± 2.37^a^	208.19^**^	<0.01
LPS(U/L)	8.73 ± 2.85^d^	11.51 ± 4.15^c^	15.09 ± 5.07^b^	27.34 ± 6.65^a^	316.33^**^	<0.01
Age, years, x ± *s*	70.17 ± 10.82^a^	69.89 ± 11.64^a^	66.27 ± 14.93^b^	61.91 ± 17.38^c^	9.92^**^	<0.01
Sex [n(%)]						
Male	62(63.26)	136(72.73)	180(68.70)	143(72.23)	2.83	0.42
Inflammatory biomarkers						
WBC (×10^9^/L)	8.52 ± 4.25^d^	9.95 ± 6.07^c^	11.55 ± 6.11^b^	13.62 ± 7.67^a^	12.68^**^	<0.01
NE (×10^9^/L)	6.81 ± 4.04^d^	7.95 ± 4.88^c^	9.73 ± 5.83^a^	11.65 ± 7.19^a^	15.63^**^	<0.01
CRP (μg/mL)	59.13 ± 52.09^d^	77.07 ± 75.71^c^	92.2 ± 77.79^b^	117.27 ± 104.71^a^	9.97^**^	<0.01
PCT (ng/mL)	2.32 ± 7.37	4.11 ± 10.51	5.77 ± 18.02	7.05 ± 14.07	2.24	0.08
Nutritional status						
Hb (g/L)	90.21 ± 20. ×10^9^/L 15	95.66 ± 21.10	97.31 ± 21.64	95.45 ± 22.52	2.15	0.09
ALB (g/L)	31.49 ± 4.51	32.11 ± 4.51	32.25 ± 3.67	31.52 ± 4.04	1.26	0.29
PA (mg/dL)	12.14 ± 5.55^a^	12.08 ± 5.59^a^	12.10 ± 5.42^a^	10.41 ± 5.18^b^	3.16^*^	0.02
RBP (mg/L)	26.500 ± 14.133^a^	21.316 ± 11.307^b^	22.630 ± 12.911^b^	21.568 ± 12.784^b^	3.09^*^	0.03
LYM (×10^9^/L)	0.96 ± 0.48	1.06 ± 1.49	0.95 ± 0.58	0.95 ± 0.55	0.72	0.54
PNI	35.66 ± 7.06^a^	35.60 ± 11.35^a^	35.74 ± 8.14^a^	33.15 ± 10.51^b^	4.43^**^	<0.01
Cytokines						
IL-2 (pg/ml)	1.22 ± 1.11	1.65 ± 2.92	1.59 ± 2.17	5.61 ± 25.72	1.43	0.24
IL-4 (pg/ml)	1.19 ± 0.90	1.68 ± 3.02	1.36 ± 1.29	1.11 ± 0.95	0.86	0.47
IL-6 (pg/ml)	55.37 ± 59.54^d^	87.82 ± 247.72^c^	253.09 ± 724.45^b^	608.80 ± 1485.86^a^	4.19^**^	<0.01
IL-10 (pg/ml)	11.62 ± 8.14	14.21 ± 21.10	42.13 ± 134.65	87.37 ± 358.95	1.67	0.18
TNF-α (pg/ml)	1.03 ± 1.15	1.22 ± 2.66	1.40 ± 1.69	0.67 ± 0.63	1.47	0.22
IFN-γ (pg/ml)	1.56 ± 1.57	1.60 ± 1.97	1.68 ± 1.84	1.08 ± 1.40	1.13	0.34

1 DAO, D-Lac, LPS: −−−.

2 DAO, D-Lac, LPS: + − −, − + −.

3 DAO, D-Lac, LPS: ++−, + − +.

4 DAO, D-Lac, LPS: +++; +, above the upper limit of the reference value range; −, within the reference value range.

^a,b,c,d^Display statistical differences between groups, if two groups have the same letter, their difference is statistically non-significant, if the different letters, their difference is statistically significant. ^**^p<0.01; ^*^p<0.05.

### 3.7 Prediction of severe intestinal barrier impairment

**Figure 5:**
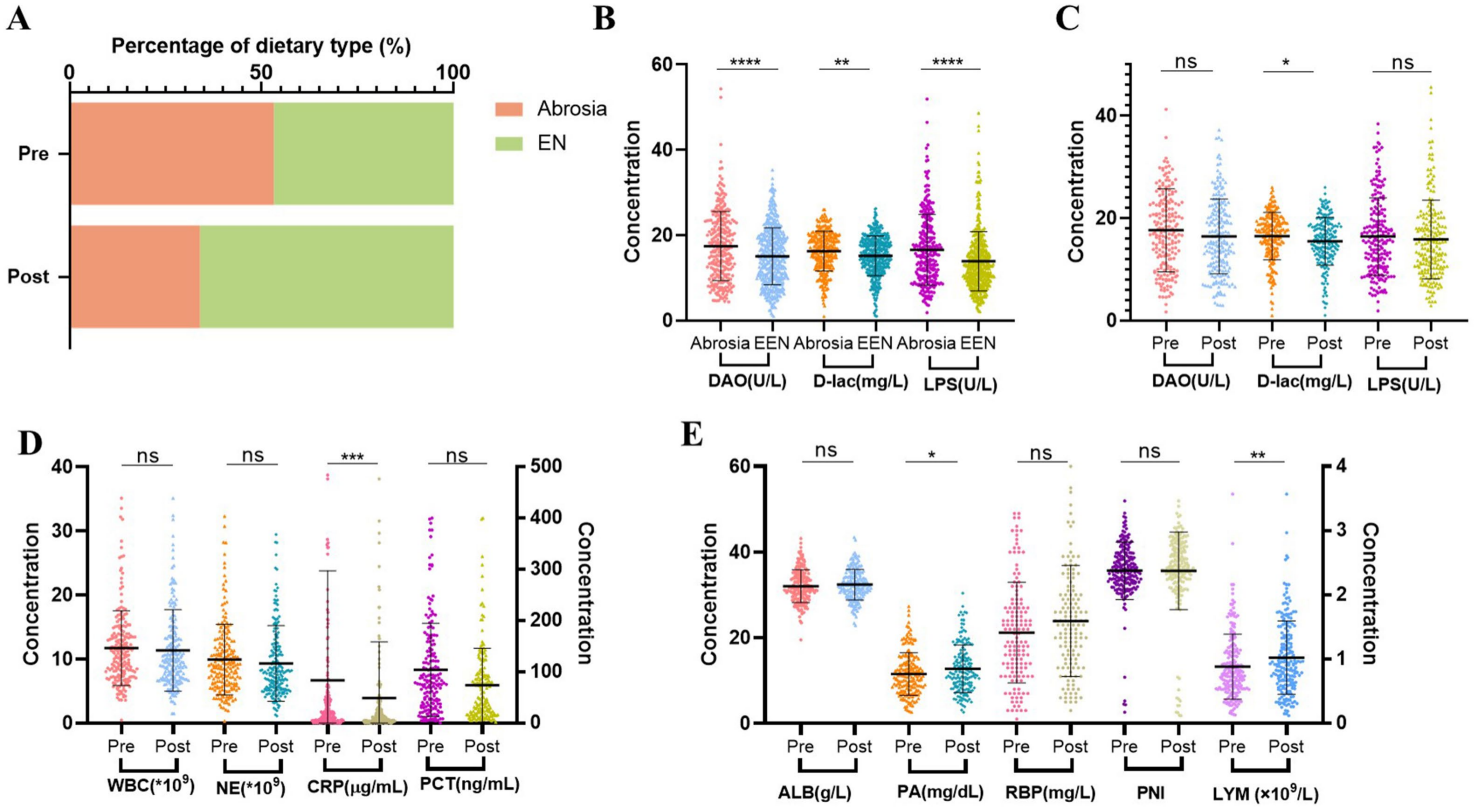
Changes after nutritional treatment for 1 week in Cohort 2. *n* = 203. **(A)** Percentage of EN in patients before and after nutritional treatment. EN, including oral diet (liquid, semi-liquid and normal-solid food), oral nutritional supplement (ONS), nasogastric tube nutrition and jejunal nutrition. **(B)** Analysis of intestinal barrier impairment in postoperative patients between no oral intake and EEN. EEN, early enteral nutrition. **(C)** Changes of intestinal barrier impairment after nutritional treatment. **(D)** Comparison of inflammation before and after nutritional treatment. **(E)** Comparison of nutritional status before and after nutritional treatment. Pre, Pre-nutritional therapy; Post, Post-nutritional therapy.

The patients were divided into 4 groups based on the degree of intestinal barrier impairment: normal group (−−−), mild impairment group (+ − − and − + −), moderate impairment group (++−, + − +), and severe impairment group (+++). There were statistically significant differences in age, WBC, NE, CRP, PA, RBP, PNI, and IL-6 among the four groups. These indicators were analyzed to predict the occurrence of severe intestinal barrier impairment (+++). And Age, WBC, NE, CRP, PA, and RBP could independently predict severe intestinal barrier impairment (+++), with corresponding areas under the curve (AUCs) of 0.61, 0.64, 0.65, 0.60, 0.60, and 0.60. Additionally, patients unable to initiate early enteral nutrition were at higher risk of severe intestinal barrier impairment, with an AUC of 0.64 (Table 3; Figures 5A,B). When these risk factors were combined to predict severe intestinal barrier impairment, the predictive performance improved, with AUC increasing to 0.71 (Table 3).

**Table 3:** Predictive analysis of severe intestinal barrier impairment.

Predictor	AUC	95%CI	Cutoff value	*p* value
Age (years)	0.609^**^	0.548–0.669	60.50	<0.01
WBC (×10^9^/L)	0.640^**^	0.583-0.697	7.75	<0.01
NE (×10^9^/L)	0.649^**^	0.592-0.706	6.73	<0.01
CRP (μg/mL)	0.602^**^	0.542-0.663	67.30	<0.01
PA (mg/dL)	0.602^**^	0.543-0.661	7.65	<0.01
RBP (mg/L)	0.599^**^	0.524–0.674	26.44	0.04
PNI	0.530	0.473–0.587		0.32
IL-6 (pg/ml)	0.561	0.462–0.661		0.22
No oral intake	0.636^**^	0.579–0.694		<0.01
Joint indicator	0.710^**^	0.660–0.760		<0.01

**p<0.01, ^*^p<0.05, Joint indicator, age + WBC + NE + CRP + PA + RBP + No oral intake.

### 3.8 Nutritional therapy and improvement in intestinal barrier impairment

The proportion of patients unable to initiate EN decreased after nutritional therapy (Figure 5A). Furthermore, the initiation of EEN in postoperative patients was closely related to intestinal barrier function. Compared to the no oral intake group, patients who started EEN promptly after surgery exhibited lower serum levels of DAO, D-lac, and LPS (Figure 5B). Moreover, nutritional therapy contributed to improvement in intestinal barrier function, primarily manifested by a reduction in intestinal permeability (Figure 5C). In addition, nutritional therapy was associated with reduced inflammatory markers, especially CRP levels. Among the nutritional indicators, PA and LYM showed notable increase compared to baseline levels, while improvements in other nutritional markers were less pronounced (Figures 5D,E).

### 3.9 Intestinal barrier impairment hindered therapeutic efficacy

**Figure 6:**
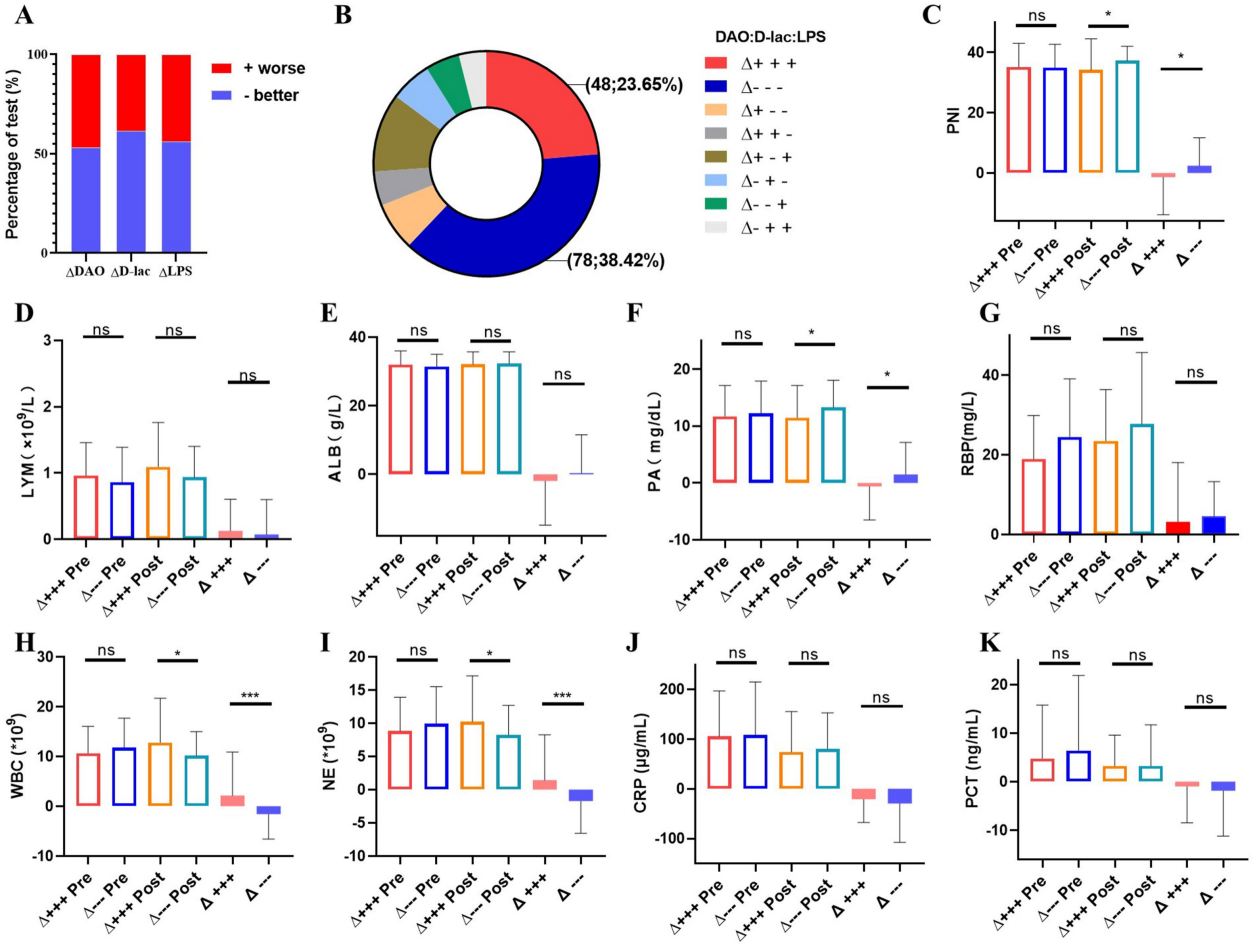
Analysis of the improvement group and the progress group of intestinal barrier impairment after nutritional treatment for1 week in Cohort 2. *n* = 203. **(A)** Changes of serum DAO, D-lac and LPS. +worse, intestinal barrier impairment worsens with increased serum DAO, D-lac and LPS; −better, intestinal barrier impairment better with lowered serum DAO, D-lac and LPS. **(B)** The number and proportion of changes in intestinal barrier function after nutritional treatment. **(C–G)** Nutritional status between the improvement group and the deterioration group of intestinal barrier function. (**H–K**) Inflammation between the improvement group and the deterioration group of intestinal barrier function. *Δ*+++, deterioration group of intestinal barrier function; Δ−−−, improvement group of intestinal barrier function; Pre, Pre-nutritional therapy; Post, Post-nutritional therapy.

Patients showed improvement in intestinal mucosal damage, intestinal permeability, and bacterial translocation after nutritional therapy, with improvement rates of 53.20, 61.58 and 56.16%, respectively (Figure 6A). Among the 203 patients that received nutritional therapy for 1 week, 38.42% (*n* = 78) showed improvement in DAO, D-lac, and LPS levels, while 23.65% (*n* = 48) had an increase in these markers, indicating a deterioration in intestinal barrier function (Figure 6B). Compared to the intestinal barrier improvement group, patients in deteriorative group had worse nutritional status, manifested by a decrease in PNI and PA levels after nutritional therapy (Figures 6C–G). Furthermore, the deteriorative group showed elevated inflammatory markers with increased WBC counts and NE levels (Figures 6H,K).

## 4 Discussion

The present study investigated the relationship between intestinal barrier function, inflammation, and nutritional status in patients after digestive surgery, providing valuable insights into the clinical implications of intestinal barrier dysfunction in the postoperative period. Our findings emphasized the high incidence of intestinal barrier impairment, primarily characterized by mucosal injury, followed by increased intestinal permeability and bacterial translocation. These results were consistent with previous studies, which have also underscored the critical role of intestinal barrier integrity in postoperative recovery (29) and its association with systemic inflammation and nutritional status.

The impairment of intestinal barrier function is closely related to the multi-layered defense mechanisms of the intestinal barrier. Our results showed that serum concentrations of DAO, D-lac, and LPS were positively correlated, with an increase in DAO preceding D-lac positivity, and bacterial translocation (LPS +) occurring only after DAO and D-lac were elevated. This sequential pattern suggests a progressive deterioration of the intestinal barrier function. Postoperative ischemia, the release of inflammatory factors, or surgical trauma can directly damage the intestinal epithelial cells and intercellular tight junctions (e.g., ZO-1 and occludin protein), leading to mucosal damage. Following mucosal injury, the permeability of the intestinal wall increases, allowing large molecular substances (D-lac) and bacterial endotoxins (LPS) to pass through the damaged mucosa into the bloodstream (30). Patients with increased intestinal permeability exhibited more severe mucosal damage, which may be related to the exacerbated oxidative stress and ischemia–reperfusion injury, such as oxygen free radicals attacking cell membrane lipids, and leading to apoptosis and necrosis (31, 32). Bacterial translocation represents the terminal stage of barrier injury, where mucosal damage and increased permeability collectively promote the passage of bacteria and endotoxins from the intestine into the systemic circulation. This process further activates systemic inflammatory responses (e.g., TNF-*α*, IL-6), forming a vicious cycle that perpetuates barrier function (33, 34).

Intestinal barrier function was closely related to the inflammation levels and nutritional status in postoperative patients. Patients with severe barrier dysfunction (+++) exhibited the highest levels of inflammatory markers and the poorest nutritional status. Furthermore, our study demonstrated that a combination of inflammatory and nutritional indicators could effectively predict severe intestinal barrier impairment, with an area under the curve (AUC) of 0.71. This predictive model provides a potential tool for early identification of high-risk patients, enabling timely interventions.

Interestingly, while elderly patients have decreased mucosal repair ability, our results showed that they exhibited less barrier damage. This paradoxical finding may be attributed to more cautious selection of surgical methods or comorbidity management in this population. Notably, elderly patients have a lower basal metabolic rate, reducing intestinal oxygen demand, thereby alleviating ischemia–reperfusion injury (35). Furthermore, aging may be accompanied by decreased glucocorticoid receptor sensitivity, reducing the destructive effects of excessive inflammatory responses on intestinal mucosa (36).

A key finding of our study was the association between early enteral nutrition (EEN) and improved intestinal barrier function. Compared with fasting patients, those who started early enteral nutrition after surgery had a lower degree of intestinal barrier impairment. This is supported by Nikniaz et al., which showed that EEN was more effective in improving postoperative nutritional status and immune indices in gastric cancer patients (37). The clinical significance of this finding lies in the potential of gastrointestinal support to safeguard intestinal barrier integrity. EEN is beneficial to intestinal barrier function recovery through multiple mechanisms. Firstly, food stimulation increases intestinal blood flow, thereby reducing ischemia–reperfusion injury. Secondly, EEN provided essential nutrients such as glutamic acid and short chain fatty acids, which promotes the proliferation of intestinal epithelial cells and the synthesis of tight junction proteins (20–23). Finally, dietary fiber promotes the colonization of probiotics while inhibiting the overgrowth of pathogenic bacteria, thereby reducing bacterial translocation (38).

Our study suggests that intestinal permeability often improves earlier during nutritional therapy. This phenomenon arises from the fact that nutrients such as glutamine (26, 27) serve as energy substrates for intestinal epithelial cells. Upon uptake, these compounds rapidly provide energy and raw materials to initiate cellular repair processes, thereby significantly restoring intestinal permeability within a short timeframe. However, mucosal repair progresses more slowly, as such injuries typically involve disruption of the intricate three-dimensional architecture of the intestinal wall. While nutritional support facilitates recovery, complete restoration of this sophisticated structural organization proves challenging in acute phases, resulting in suboptimal clinical improvement. Furthermore, bacterial translocation presents additional therapeutic hurdles due to its multifactorial pathogenesis involving microbial overgrowth, barrier dysfunction, and systemic immune dysregulation. Although nutritional interventions partially ameliorate epithelial barrier defects, they struggle to concomitantly modulate gut microbiota composition and fully restore lymphatic surveillance or immune homeostasis, ultimately limiting their efficacy in controlling bacterial translocation.

Unfortunately, a subset of patients still experienced a continued deterioration of intestinal barrier function with increased serum levels of DAO, D-lac and LPS. Compared to the group with improved intestinal barrier function, these patients showed worsening nutritional and inflammatory indicators despite receiving nutritional treatment. This suggests that deterioration of the intestinal barrier may trigger a vicious cycle of inflammation and poor response to nutritional treatment, emphasizing the importance of close monitoring and timely intervention to prevent the progression of intestinal barrier dysfunction.

Our results revealed a complex interplay between intestinal barrier injury markers (DAO, D-lactate, LPS) and cytokine dynamics. Elevated serum levels of DAO, D-lac and LPS were strongly correlated with increased IL-6, suggesting that intestinal epithelial damage promotes pro-inflammatory responses. Notably, LPS levels exhibited a positive association with IL-6, reinforcing its role in activating Toll-like receptor 4 pathways and subsequent cytokine storms (39). Conversely, IL-10 demonstrated an inverse relationship with DAO, implying compensatory anti-inflammatory mechanisms during barrier repair.

This study has some limitations that warrant attention in future research. First, the retrospective observational design of this study, while useful, are inherently limited by recall bias, selection bias, and potential confounding due to reliance on pre-existing data not collected for research purposes, and cannot establish causality. Second, the current study has merely delineated this epidemiological pattern, while the underlying molecular mechanisms remain to be elucidated. Finally, the nutritional therapy in this study consisted of comprehensive supplementation with macronutrients and essential micronutrients; however, the effect of each individual nutrient component was not examined. Further studies could investigate the effects of individual nutrients on intestinal barrier function to provide more targeted and effective nutritional intervention strategies.

## 5 Conclusion

Our study provides comprehensive evidence on the relationship between intestinal barrier function, inflammation, and nutritional status in patients who underwent digestive surgery. The findings highlight important implications for clinical practice in terms of early detection, prevention, and treatment of intestinal barrier dysfunction and its related complications.

## Data Availability

The raw data supporting the conclusions of this article will be made available by the authors, without undue reservation.
